# Vestibular Schwannoma Presenting with Bilateral Papilledema Without Hydrocephalus: Case Study

**DOI:** 10.7759/cureus.1862

**Published:** 2017-11-20

**Authors:** Carlos Candanedo, Samuel Moscovici, Joshua M Kruger, Cezar J Mizrachi, Ruth Eliahou, Sergey Spektor

**Affiliations:** 1 Neurosurgery, Hadassah-Hebrew University Medical Center; 2 Ophthalmology, Hadassah-Hebrew University Medical Center; 3 Radiology, Hadassah-Hebrew University Medical Center

**Keywords:** acoustic neuroma, hydrocephalus, papilledema, vestibular schwannoma, pseudotumor cerebri

## Abstract

Bilateral papilledema secondary to obstructive or communicating hydrocephalus in patients with vestibular schwannomas is a known presentation; however, papilledema in the absence of hydrocephalus is rarely reported and its mechanism is poorly understood. We report a case of a 20-year-old woman presenting with visual deterioration and bilateral papilledema on fundoscopy. Magnetic resonance imaging (MRI) revealed a giant vestibular schwannoma with no sign of hydrocephalus. The only imaging evidence of increased pressure on preoperative imaging studies was seen on a T2-weighted MRI, where there was subtle dilatation of the arachnoid space of the optic sleeve. We presume that this patient developed papilledema by some mechanism not connected to hydrocephalus. In a young patient, papilledema may be a sign preceding hydrocephalus, or she may have had pseudotumor cerebri concomitant with her vestibular schwannoma. In either case, removal of the vestibular schwannoma solved the problem. She had complete visual recovery, irrespective of the mechanism.

## Introduction

Vestibular schwannoma accounts for approximately 8% of all intracranial tumors and represents the most common neoplasm of the cerebellopontine angle (CPA) [1-3]. Typical presenting symptoms are well known, including those related to brainstem compression and due to obstructive hydrocephalus [1, 4]. Papilledema is an uncommon finding, occurring in about 8% of patients with vestibular schwannoma [5]; however, papilledema in the absence of hydrocephalus is rarely reported. We present our experience with a patient with a giant vestibular schwannoma who manifested with visual deterioration caused by bilateral papilledema without hydrocephalus, a review of previously reported cases, and consider the possible etiology of this unusual presentation.

## Case presentation

**Figure 1:**
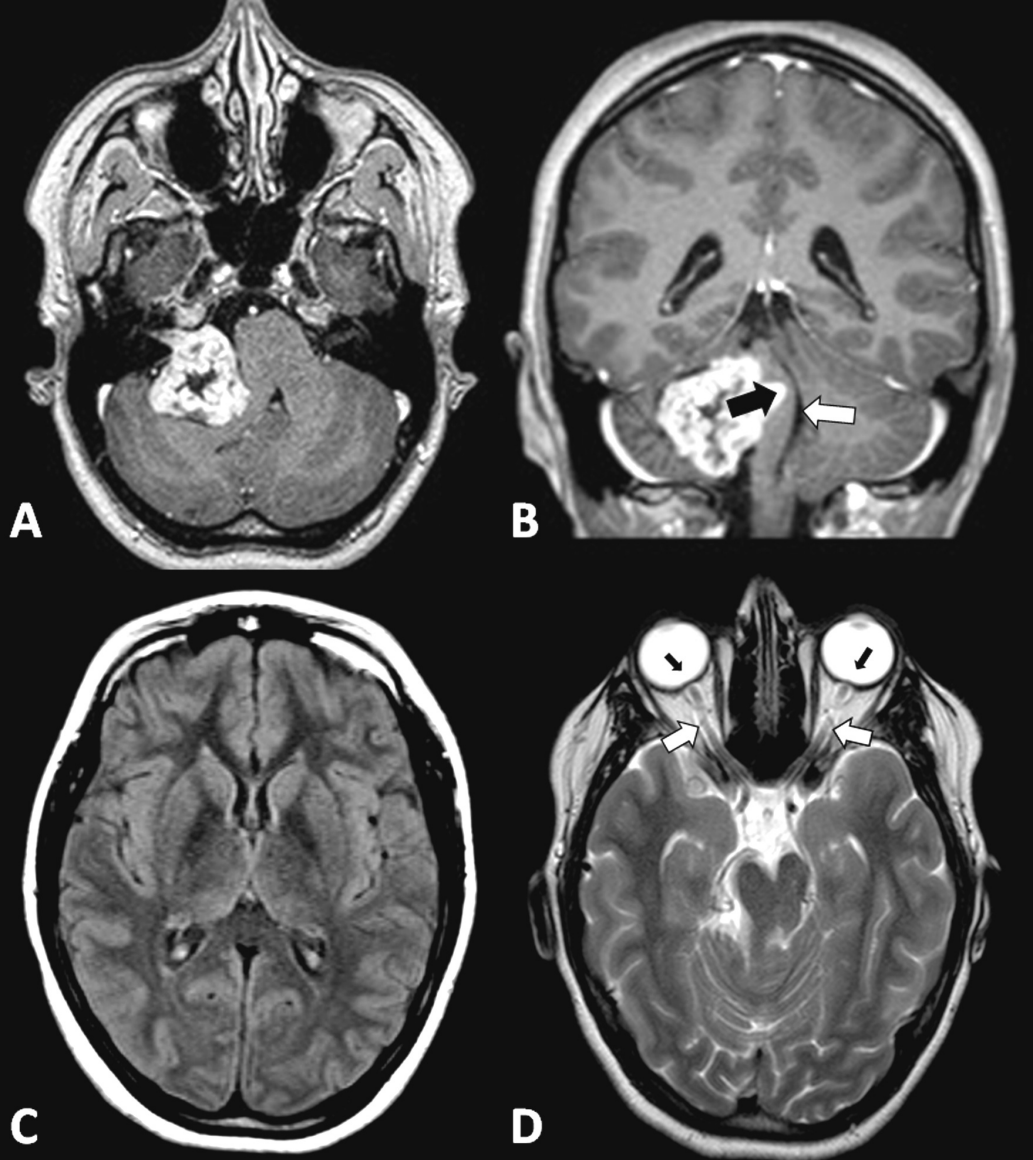
Preoperative magnetic resonance imaging (MRI) (A) Axial T1-weighted gadolinium-enhanced MRI showing a sharply marginated non-homogeneously enhancing right cerebellopontine angle (CPA) mass extending into the right internal auditory canal in a 20-year-old woman. (B) Coronal T1-weighted gadolinium-enhanced MRI showing compression on the brainstem from tumor mass effect (black arrow), with a patent aqueduct of Sylvius (white arrow). (C) Axial T2-weighted fluid-attenuated inversion recovery (FLAIR) MRI showing normal ventricle size without signs of transependymal edema. (D) Axial T2-weighted MRI. The hyperintense signal surrounding the optic nerves (white arrows) is due to increased CSF pressure leading to dilatation of the paraneural subarachnoid space. Note flattening of the posterior aspect of the globes (black arrows).

**Figure 2:**
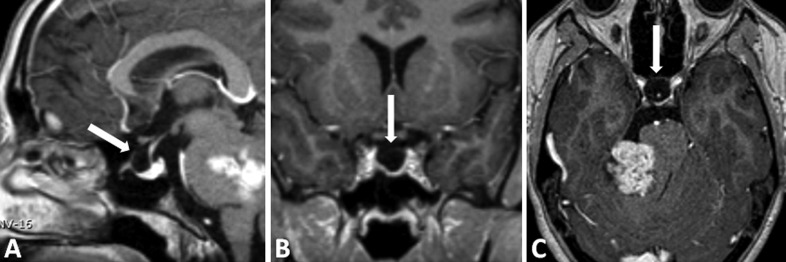
Preoperative magnetic resonance imaging (MRI) (A) Sagittal, (B) coronal, and (C) axial T1-weighted gadolinium-enhanced MRI in the same patient revealed an enlarged sella relative to a normal pituitary gland.

A 20-year-old female, otherwise healthy, presented for ophthalmological evaluation due to a one-month history of decreasing visual acuity and transient episodes of darkened vision bilaterally. The examination revealed bilateral papilledema. Magnetic resonance imaging (MRI) demonstrated a giant (40 mm diameter) enhancing cerebellopontine angle (CPA) tumor extending into the right auditory canal, characteristically compressing the brainstem, Hannover class T4a (Figure 1A) [6]. There was no sign of hydrocephalus and the aqueduct of Sylvius was patent; however, on the T2-weighted study, a hyperintense signal surrounding the optic nerves bilaterally revealed subtle dilatation of the arachnoid space of the optic sleeve and the posterior aspect of the globes was flattened (Figure 1B-D). The sella turcica was rather enlarged as seen in Figure 2.

The patient was referred for neurosurgical evaluation. She reported decreased hearing, dizziness, imbalance, unstable gait, and headache of several weeks duration but denied any incontinence or cognitive symptoms. On physical examination, there was right facial hypoesthesia, right peripheral facial palsy (House-Brackmann Grade 2 (HB2)), right beating nystagmus, diadochokinesis (DDK), positive Romberg sign, and dysmetria in the right extremities [7]. A lumbar puncture was not performed due to the risks with a large mass in the posterior fossa and papilledema.

**Figure 3:**
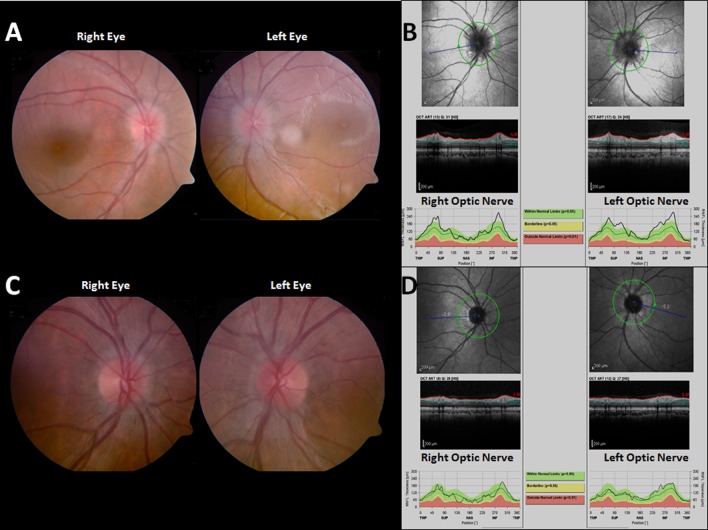
Pre- and postoperative neuro-ophthalmological examinations (A) Preoperative fundoscopy revealed bilateral blurred optic disc margins. (B) Preoperative optical coherence tomography (OCT) of the parapapillary zone demonstrates thickening of the retinal nerve fiber as a result of swelling. (C) Fundoscopy and (D) OCT performed three months after surgery revealed complete  resolution of the papilledema.

Visual acuity was 6/12 bilaterally. Fundoscopy confirmed bilateral papilledema with normal visual fields (Figure 3A). On optical coherence tomography (OCT), there was bilateral thickening of the retinal nerve fiber layer (Figure 3A).

Surgical procedure

The tumor was resected via a right retrosigmoid approach. The facial nerve was very adherent in the premeatal area; thus, a 10 × 10 mm “fingernail” residual was left to ensure facial nerve preservation in this young woman. The surgery was uneventful.

Postoperative course

Facial nerve function was unchanged (HB2) following surgery. Postoperative head CT showed favorable resection with no sign of significant bleeding or hydrocephalus. The early and late postoperative course was uneventful except for a significant pseudomeningocele in the surgical wound. The patient was discharged home with ambulatory physiotherapy because of mild ataxia. The pseudomeningocele had contracted significantly and her ataxia improved gradually.

Follow-up

At the three-month follow-up examinations, her visual acuity was normal (6/6), the papilledema had subsided on fundoscopy, and the thickness of the retinal nerve fiber layer had normalized bilaterally on OCT (Figure 3B).

**Figure 4:**
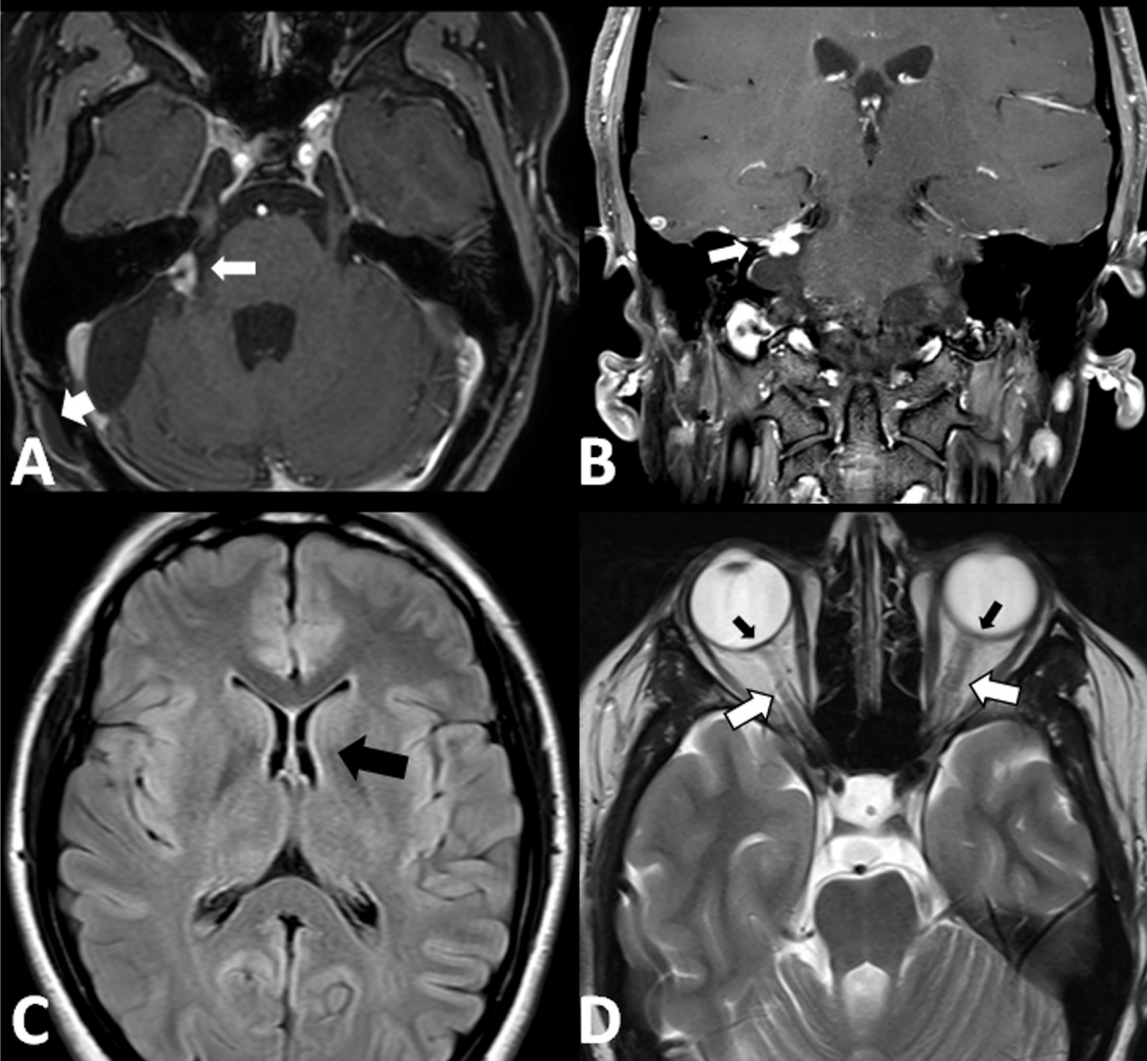
Magnetic resonance imaging (MRI) at two-year follow-up MRI performed two years after resection. (A) Axial T1-weighted gadolinium-enhanced image showing a 10 × 10 mm “fingernail” residual of the vestibular schwannoma (arrow) and persistent pseudomeningocele (arrowhead). (B) Coronal T1-weighted gadolinium-enhanced MRI. The residual is seen (white arrow). (C) Axial T2-weighted fluid-attenuated inversion recovery (FLAIR) image showing normal ventricle size. (D) Axial T2-weighted image shows marked bilateral reduction in the dilatation of the subarachnoid space within the dural sleeve surrounding the optic nerves (white arrows) and normal curvature of the globes (black arrows).

MRI performed three months after surgery showed a small residual plaque of tumor. There were no signs of hydrocephalus. On T2-weighted MRI, dilatation of the arachnoid space of the optic sleeve was markedly reduced and the globes had regained their normal curvature (Figure 4). No changes in the residual tumor were seen on the two-year follow-up MRI.

## Discussion

Papilledema is a known sequela of elevated intracranial pressure (ICP), which generally develops due to obstructive hydrocephalus, venous obstruction, supratentorial mass effect, or pseudotumor cerebri. In spite of the large tumor diameter with significant brainstem compression, the aqueduct of Sylvius appeared patent and the ventricles were of normal size. The only imaging evidence of increased pressure was seen on a T2-weighted MRI, where there was dilatation of the arachnoid space of the optic sleeve and flattening of the posterior aspect of the globes (Figure 1D).

Bilateral papilledema in this young patient may have been a relatively early sign preceding ventricular dilatation. Pressure on the brainstem would have produced a tendency for the aqueduct to occlude, with resulting resistance to normal CSF flow. ICP elevation could have served as a mechanism to maintain aqueduct patency in this case, as evident in the coronal view (Figure 1B).

Harada, et al. described a 26-year-old patient with neurofibromatosis Type 2 (NF2) who presented with bilateral vestibular schwannomas associated with bilateral optic disc edema [8]. There was aqueduct obstruction without hydrocephalus. The authors suggest that ventricular obstruction may be sufficient to raise intracranial pressure, while not necessarily enlarging the ventricles beyond the normal range in a young patient.

More recently, Grainger and Dias [9] and Matos, et al. [10] suggested that protein secretion into the CSF by vestibular schwannomas may cause an impaired reabsorption of CSF, albeit intermittently, resulting in a type of communicating hydrocephalus. The same mechanism was proposed in patients with spinal schwannomas and normal pressure hydrocephalus [8]. Harada also considered high protein concentration as the possible mechanism in his NF2 patient. Since our patient presented with normal ventricles, we didn’t measure perioperative CSF protein concentration.

There was a documented resolution in the papilledema after tumor resection. Regardless of the mechanism, since the tumor was removed soon after the patient’s vision was compromised and before there was permanent damage to the optic nerve, we were not only successful in treating the papilledema, but also in facilitating recovery to normal visual acuity. We can speculate that there are two possible mechanisms here: papilledema may precede ventricular dilatation, regardless of whether it is of obstructive or absorptive origin, or the young woman could have two concomitant diseases, a vestibular schwannoma that became enlarged on the background of a formerly silent pseudotumor cerebri syndrome, causing its decompensation. It may well be that elevated intracranial pressure (ICP) contributed to the development of the postoperative pseudomeningocele, which could have played a positive role in replacing and obviating the need for a CSF diversion procedure.

## Conclusions

A 20-year-old woman presented with visual deterioration and bilateral papilledema on fundoscopy. Her MRI was characteristic for a giant vestibular schwannoma without signs of hydrocephalus. The papilledema was permanently resolved after surgery. This illustrates the possibility that visual decline and severe papilledema may be manifestations of a giant vestibular schwannoma without the signs of hydrocephalus. Another possible explanation is a mechanism similar to pseudotumor cerebri or a preexisting silent pseudotumor cerebri syndrome.
